# Multimodal prehabilitation for frail older adults having hip or knee replacement: a qualitative exploratory study of barriers and facilitators

**DOI:** 10.1186/s12891-026-09803-z

**Published:** 2026-04-20

**Authors:** Emma Johnson, Marcus Jepson, Vikki Wylde

**Affiliations:** 1 Bristol Medical School, University of Bristol, Bristol, UK; 2 National Institute for Health and Care Research Bristol Biomedical Research Centre, University Hospitals Bristol and Weston NHS Foundation Trust and University of Bristol, Bristol, UK

**Keywords:** Prehabilitation, Frailty, Exercise, Nutrition, Knee replacement, Hip replacement, Qualitative methods

## Abstract

**Background:**

Frailty is common in patients undergoing joint replacement, with approximately 20–25% of patients having moderate-severe frailty. In an orthopaedic context, frailty is associated with increased mortality, longer hospital stays, post-operative complications, more hospital admissions, and worse pain and function after surgery. Multimodal prehabilitation may improve outcomes for frail patients undergoing joint replacement, however evaluation of the effectiveness of this approach is needed. Prior to conducting a randomised controlled trial (RCT), a feasibility study can address key uncertainties and explore how to optimise trial design and delivery. The aim of this qualitative study embedded within a randomised feasibility study was to gain an understanding of the experiences of older frail patients waiting for a total hip replacement (THR) or total knee replacement (TKR) participating in a multimodal prehabilitation programme, and to seek insight into the barriers and facilitators influencing adherence.

**Methods:**

This was a qualitative study embedded within a randomised feasibility study of a 12-week daily home-based prehabilitation programme (exercise and protein supplements) for frail patients aged ≥ 65 years on the waiting list for primary THR or TKR. Participants in the feasibility study were invited to participate in a semi-structured qualitative interview. Interview questions were informed by a topic guide, and an inductive thematic analysis of interview transcripts was conducted.

**Results:**

Interviews were conducted with 10 participants randomised to the intervention group. Three overarching themes that influenced adherence to the prehabilitation programme were identified. Intervention design factors included the intervention appointment and telephone support, provision and clarity of written information and tailoring of the exercise programme. Internal factors included pain, general health and wellbeing, and personal motivations and perceived value. Environmental/external influences included social influences and daily life (integration, interruption and changes to usual routine).

**Conclusions:**

This qualitative study identified multifactorial barriers and facilitators that influence frail older adults’ adherence to multimodal prehabilitation before joint replacement. Our findings also highlight the importance of provision of tailored prehabilitation and additional support and resources to optimise accessibility and adherence.

**Trial registration:**

ISRCTN11121506, registered 29 September 2022.

**Supplementary Information:**

The online version contains supplementary material available at 10.1186/s12891-026-09803-z.

## Background

Total hip replacement (THR) and total knee replacement (TKR) are two of the most common elective surgeries performed in the National Health Service (NHS), with over 200,000 undertaken every year [1]. The main indication for these procedures is osteoarthritis, a painful condition which increases with age, and the average age of patients undergoing these operations is 69–70 years [1]. Frailty, a clinical state marked by the progressive and gradual decline in physical, psychological and social functions is also associated with the ageing process [2]. It is common amongst older people awaiting joint replacement, with approximately 40–45% having mild frailty and 20–25% having moderate-severe frailty [3], assessed using the electronic Frailty Index with severity categories based on increasing risk of mortality, hospitalisation and nursing home admission [4]. Older frail patients are particularly vulnerable to experiencing poorer outcomes from their surgery, including increased mortality, longer hospital stays, post-operative complications, more hospital admissions, and worse pain and function [3, 5–11].

Frailty is a potentially modifiable condition, with physical inactivity and inadequate nutrition being key contributing factors that can be targeted for intervention [12]. Increasing physical activity and protein intake offers benefits to those living with the condition [13–16]. Prehabilitation improves function and reduces length of hospital stay and post-operative complications for frail patients undergoing surgery for cancer and elective abdominal surgery [17–19]. Although multimodal prehabilitation could benefit older frail orthopaedic patients, evaluation of the effectiveness in this population is needed. However, prior to conducting a randomised controlled trial (RCT), a feasibility study is necessary to understand how to optimise the design of the trial and intervention. Embedding qualitative methods within a feasibility study can provide insight into the contextual factors that influence adherence, highlight barriers and facilitators to behaviour change and the experiences of participants and their responses to the intervention [20, 21]. Utilisation of qualitative methods have particular merit within the current area of study as previous research suggests that adherence to interventions amongst older patients with frailty may be suboptimal [22, 23]. The aim of this qualitative study was to gain an understanding of the experiences of older frail patients waiting for a THR or TKR participating in a multimodal prehabilitation programme, and to seek insight into the barriers and enablers influencing enactment.

## Methods

### Design

This qualitative interview study was embedded in the Joint PREP study. Joint PREP was a feasibility study for a RCT to evaluate the clinical and cost-effectiveness of prehabilitation for frail patients undergoing THR or TKR. Full details of the study design and methods are reported elsewhere [24, 25]. Eligibility criteria included ≥ 65 years of age, waiting for primary THR or TKR and frail (defined as score of ≥ 4 on the self-reported Groningen Frailty Indicator (GFI) [26], an accepted threshold for defining frailty [27]). Sixty-four participants from three NHS hospitals were randomly allocated on a 1:1 ratio to the intervention or usual care group. Those assigned to the intervention group attended a 1:1 appointment with a trained physiotherapist to develop a tailored exercise programme. They were asked to follow the programme at home for 12-weeks before their operation and were provided with two booklets to assist them with the exercises. Participants were also supported by six telephone calls from a physiotherapist. In addition, the intervention group were provided with a protein supplement, in the form of jelly pots or protein powder (to make into shakes). They were asked to consume the supplement daily, within 3h after exercising, over the same 12-week period. Further details of the prehabilitation programme are provided in Table 1. Reporting of the study follows the COnsolidated criteria for REporting Qualitative research (COREQ) guidance and a completed checklist is provided in the supplementary materials (Additional file 1).

**Table 1:** Summary of intervention

Protein	Exercise
• Participants asked to consume 20 g of additional protein each day for up to 12-weeks prior to their planned / approximated surgery date. • Choice of two protein options: ProSource jelly pot (choice of flavours) or Protein powder (Vanilla or Chocolate flavour). • Participants provided with instructions for eating the jelly pots or making up and drinking the powder shakes. • Participants asked to consume the protein, if possible, within three hours after exercise (to match peak of muscle protein synthesis) and between meals (to limit impact on appetite).	• Participants asked to undergo 12-week personalised home-based exercise intervention with regular contact/support. • Exercise programme tailored to each participant by a trained physiotherapist during 1:1 in-person appointment at the beginning of the intervention period. • Booklets provided to participants to assist them with completing the exercises. Written information included the instructions/illustrations for each of the exercises. • Where appropriate, participants issued with an exercise step and/or gym ball and/or TheraBand (at their initial appointment) to be used according to abilities. • Participants supported by telephone calls (or videoconference) from trained physiotherapist after approximately 1, 2, 4, 6, 8, and 10 weeks.

### Recruitment and sampling

**Fig. 1:**
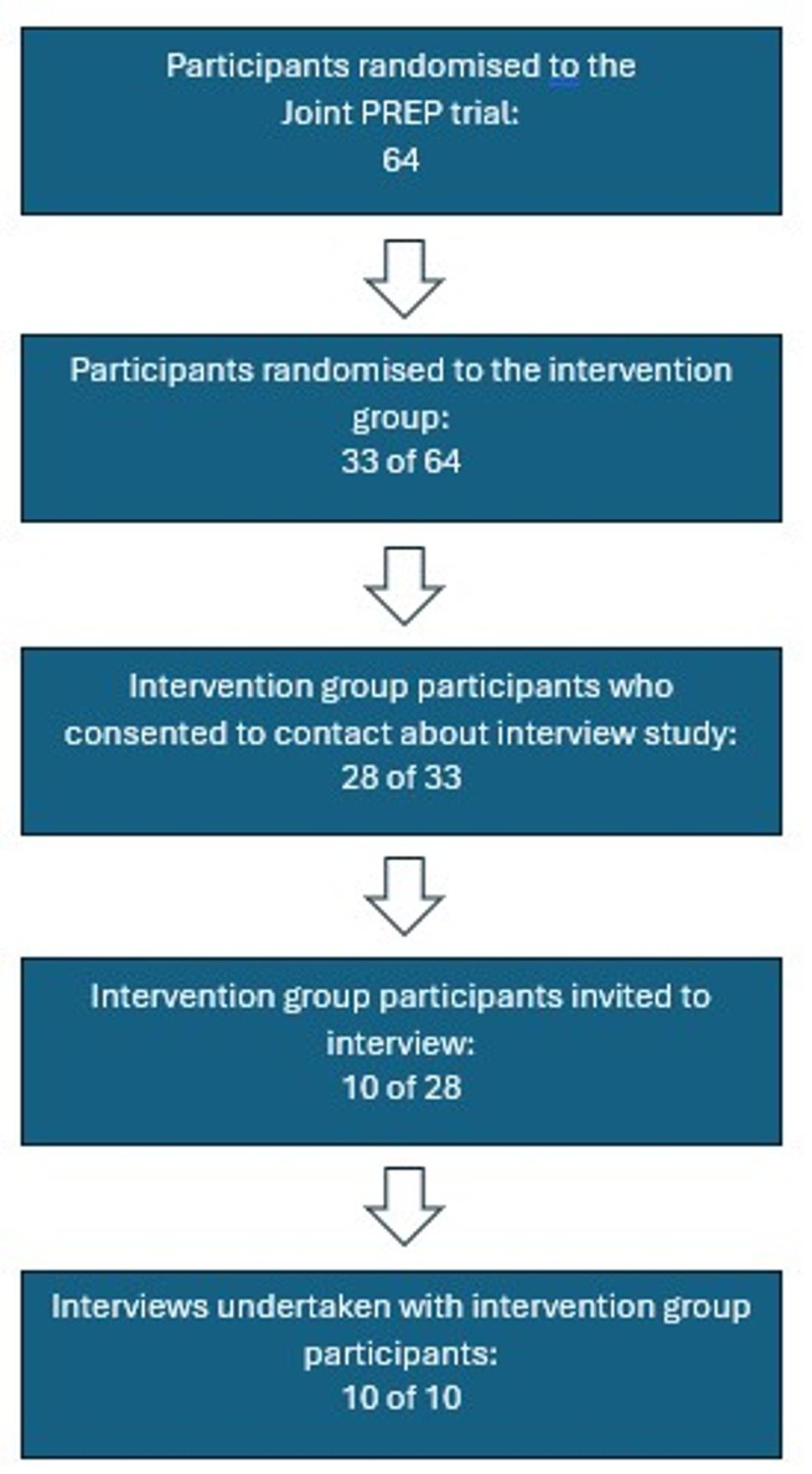
Participant recruitment into the interview study

Patients on a waiting list for THR or TKR at three NHS hospitals were sent a screening questionnaire and information pack about the Joint PREP study. Eligible patients provided informed written consent prior to participation. As part of this process, patients were asked if they were willing to be approached to take part in an interview. Of the 64 randomised participants, 49 participants agreed to an interview, 28 of whom were randomised to the intervention group (see Fig. 1). The contact details of those who gave their consent to the interview study were given to the qualitative researcher by the site research teams, who then contacted participants by telephone to introduce herself (which included the sharing of information about her role on the project and research background), discuss the aims of the interview, answer any questions and to arrange a suitable time and mode for their interview. Our aim was to interview 15–20 randomised participants (intervention/usual care), with precise numbers driven by data saturation. Of those intervention group participants approached for the one-off interview (*N* = 10), all agreed to proceed to interview. Purposive sampling guided the selection of trial participants to ensure representation of men and women, patients who were a range of ages, those from across trial sites, operated joint and trial arms.

### Data collection

Interviews were undertaken over a 6-month period between May and October 2023 by a qualitative researcher with prior experience of interviewing patients with musculoskeletal conditions (EJ). Interviews were conducted with participants randomised to the intervention group after they had completed the intervention, and with participants randomised to the usual care group after they had been informed of their group allocation. Interview questions were informed by a semi-structured topic guide which aimed to elicit experience of trial participation, including willingness to be randomised and views on questionnaire completion. For those in the intervention group, questions also focused on experiences of the intervention appointment and factors influencing participants willingness to engage with the intervention. Refinements were made to the topic guide during data collection in response to novel areas of interest or relevance identified during early interviews and probes were used to facilitate elaboration and to achieve depth. The interview topic guide is provided in the supplementary material (Additional file 2). The qualitative researcher took field notes immediately after each interview with the purpose of documenting contextual information and recording important features of the interview.

### Data analysis

Audio files were transcribed verbatim, checked for accuracy against the original recordings and anonymised. Transcripts were uploaded, along with the interview notes, to Nvivo (Version R1, QSR International Pty Ltd., Doncaster, Australia). Our approach to interrogating the interview data was informed by thematic analysis. We applied an inductive thematic approach to analysis, with data coded into themes and sub-themes. Data collection and analysis were carried out in parallel after the first four interviews, allowing emergent themes to alter the coding as the analysis progressed [28]. Two authors (EJ & MJ) met on several occasions to discuss, revise and agree the development of these themes.

### Reflexivity

To ensure the credibility of findings and interpretations, the first author (EJ), who undertook all interviews and led on analysis, kept a diary during the data collection and analysis period. This served as an aid to reflexivity, facilitating self-reflection on her position as a post-doctoral, white, non-frail woman in the research process and presentation of findings, and was an opportunity to both acknowledge and challenge her pre-conceptions and assumptions. In the presentation of findings, we have aimed to avoid demonstrating any bias by presenting quotations verbatim from our participants.

### Patient and public involvement

This study benefited from the involvement of PEP-R (the Patient Experience Partnership in Research group), an established forum of seven patients who have had, or are having treatment for musculoskeletal health conditions, including joint replacement. PEP-R met regularly to discuss Joint PREP, and provided input into the design, delivery, interpretation and dissemination of the study. For the nested qualitative work this included helping to design the interview topic guide and providing input into, and feedback on, the interpretation of themes identified within the data set. This provided additional insights from the patient perspective as research participants were not themselves invited to provide feedback on findings.

### Ethics

Research ethics committee approval for the main Joint PREP study, including this qualitative component, was obtained from the East of Scotland NHS Research Ethics Committee 2 on the 30th of August 2022 (REC reference 22/ES/0033) and Health Research Authority approval on the 6th of September 2022.

## Results

### Participants

Nineteen people agreed to take part in the interview study; of these 10 were randomised to the intervention group, 7 were randomised to the usual care group and 2 declined participation in the feasibility study but consented to take part in an interview. Broader findings from the qualitative study for all participants have been reported separately as part of the findings from the Joint PREP feasibility study [25]. This article focuses on developing an understanding of the factors that influenced adherence to the intervention, and therefore data presented here is based on the analysis of the interviews with the 10 participants who were randomised to the intervention group. Demographics of these 10 participants are presented in Table 2. Interviews, which lasted between 25 and 100 min (average 53 min), were conducted over the telephone (*n* = 9) or in the participants’ home (*n* = 1). The participant who was interviewed at home was alone during the interview; however, we do not know if this was the case for those who we spoke to over the telephone.

**Table 2:** Participant demographics

Participant identifier	Gender	Age	Ethnicity	Relationship status	Living situation	Occupation	Joint	GFI score^a^
Ppt 7	Female	83	White	Widowed	Lives alone	Retired	Hip	4
Ppt 8	Female	74	White	Unknown	Unknown	Retired	Knee	7
Ppt 12	Female	85	White	Widowed	Lives alone	Retired	Hip	4
Ppt 13	Female	79	White	Married	Lives with husband	Retired	Knee	6
Ppt 14	Female	78	White	Married	Lives with husband	Retired	Knee	6
Ppt 15	Male	83	White	Single	Lives alone	Retired	Hip	6
Ppt 16	Female	65	White	Widowed	Lives alone	Retired	Hip	7
Ppt 17	Male	70	White	Married	Lives with wife	Retired	Hip	4
Ppt 18	Female	71	White	Married	Lives with husband	Retired	Knee	5
Ppt 19	Female	71	White	Married	Lives with husband	Retired	Hip	5

^a^The GFI is scored 0–15, with a higher GFI score indicating a greater level of frailty

### Contextual information and reflections on participants engagement with the intervention

To understand participants experiences and views, it is important to acknowledge that most had been on a waiting list for joint replacement for a long time, often several years. At the time of interview, the lives of many had become increasingly restricted and participants spoke of experiencing constant pain, issues with mobility and a reliance on aids and equipment (e.g. sticks and stair lifts). It was common for participants to have multiple co-morbidities, and some revealed that they depended on family and friends, or paid help, to support them in their daily lives.

Variation existed amongst participants with regards to their commitment to, and engagement with, the exercise programme. Although the majority reported that they had attempted the exercises on most days over the 12-week period, a small number did break from the regimen for a more extended time or discontinued the treatment altogether. Many participants reported that when undertaking the routine, they did not execute all recommended exercises and/or the suggested number of repetitions. Although some found the exercises easier to do over time, this was not the case for everyone. Most participants reported that they had been willing to consume the protein supplement on the majority of days over the 12-week intervention period, with only three revealing that they had stopped taking the protein supplement before the end of the intervention period. Nearly half adhered to the request to consume the supplement within 3-hours of undertaking the exercise programme, the remainder chose to take it at a time of day more convenient to them. Details of participants’ adherence to the prehabilitation programme and reasons for stopping the intervention altogether, where relevant, are outlined in Table 3.

**Table 3:** Record of adherence to prehabilitation programme

Participant identifier	Adhered to exercise programme?^a^	Reasons disclosed during interview for non-adherence to exercise programme	Adhered to protein supplements? ^b^	Reasons disclosed during interview for non-adherence to protein supplement
Ppt 7	Yes		Yes	
Ppt 8	Yes		Yes	
Ppt 12	Yes		Yes	
Ppt 13	Yes		No	Taking protein supplement felt like a punishment - longstanding issues with swallowing.
Ppt 14	Did not return adherence log	Unwilling to re-engage following hospital stay during intervention period– complex factors including pain and fear.	Did not return adherence log	Discontinued protein supplement during hospital stay during intervention period– unwilling to re-engage on discharge.
Ppt 15	Yes		Yes	
Ppt 16	Did not return adherence log		Did not return adherence log	
Ppt 17	No	Stopped after taking a break from the exercise routine whilst away on a 2-week holiday. On return home struggled to re-engage with the regimen and subsequently chose to stop altogether (pain contributing factor).	No	Discontinued protein supplement as had chosen to disengage from the exercise intervention and saw little point in engaging with this component.
Ppt 18	Yes		Yes	
Ppt 19	Yes		Yes	

^a^Adherence to the exercise programme was defined as completing the exercises ≥ 3 days per week for at least 10 weeks (or 80% of intervention duration if the time available was shorter than 12 weeks because surgery occurred earlier than expected)

^b^Adherence to the protein supplements was defined as consuming the protein supplement on ≥ 4 days per week for at least 10 weeks (or 80% of intervention duration if the time available was shorter than 12 weeks because surgery occurred earlier than expected)

Three overarching themes were identified which helped to shape understanding of key factors that influenced participants experiences and adherence to the prehabilitation programme over the 12-week period: (1) Intervention design factors; (2) Internal factors and (3) Environmental/external influences.

### Theme 1: Intervention design factors

#### Intervention appointment and telephone support

Participants attended an appointment with a trained physiotherapist at the hospital, where the intervention was introduced. They were amenable to attending the appointment as it was viewed as a worthwhile investment, an opportunity to receive guidance from a physiotherapist, something that had been lacking for many during the pre-operative period. The in-person format, which enabled participants the opportunity to be shown, and to practice, the exercises in front of the physiotherapist, was viewed as imperative as it offered reassurance and enhanced understanding of what they were expected to do.

*… it was very helpful going in and seeing her […] she demonstrated everything which was very helpful […] it’s all very well giving me these exercises but unless you’re doing them properly you probably could do more damage. But [name of physio] explained and showed me and then watched me do it which*,* you know*,* was very helpful and I knew I was doing it properly then. (Ppt 12)*

The 1:1 mode of delivery was seen as advantageous over group delivery, as it meant that the exercise programme could be personalised to a participant’s capabilities. It was also viewed as helpful in establishing trust between patient and practitioner.

*… my regimen was tailored. You don’t go on to do that one*,* you stick to this one […] So that’s why the one to one is so important. […] I think the personal one to one is one of the most important things because I know it sounds silly but it’s a question of trust as well isn’t it*,* because you know if you’re not quite sure you can turn to somebody that you’ve had a conversation with*,* that you’ve been able to ask questions. To take that away I think would harm if you put it out as a long-term thing. (Ppt 13)*

Following the initial appointment, participants were supported by 6 follow-up telephone calls from the physiotherapist over the next 12-weeks. They expressed how the telephone calls had helped to sustain their motivation and engagement with both components of the intervention and had been a valuable opportunity to ask questions, amend their exercise programme and receive reassurance that they were doing everything correctly. The continuity of receiving the telephone calls from the physiotherapist who they had met during the initial appointment was viewed as advantageous, as rapport and trust had already been built, helping to negate the need to meet in person again.

*… they [the phone calls] were very positive and gave you the confidence that you were doing it right […] I found having [name of physio]*,* because we’d had a face to face*,* I found that quite reassuring. (Ppt 7)*

*…keeps you motivated because you know you’re going to get a call and you know*,* yeah makes you do it knowing that somebody’s going to call you back over it. (Ppt 18)*

Although most viewed the timing and frequency of the calls as appropriate, one participant suggested that the regularity and timing of support could be tailored based on a patient’s needs and confidence level.

*The spacing was fine yeah. I think perhaps I mean depending on the person […] if you have this initial meeting*,* I’m sure in your work you can judge whether a person is vulnerable or insecure […] somebody might need that weekly for reassurance more than anything […] once a fortnight was fine for me. (Ppt 13)*

#### Provision and clarity of written information

Participants received a booklet which contained instructions and illustrations for each of the exercises. Responses suggest that the ‘clear’ and ‘easy to follow’ content was helpful, particularly in the initial weeks, in assisting participants to recall which exercises they had to do and exactly how they should be executed.

*[The physio] kitted me out with a leaflet which I found very helpful […] I could look at it and do exactly what the picture showed to do […] I had to keep looking at it and sort of thinking am I doing it right. But in the end*,* I could do it automatically and I knew exactly what to do. (Ppt 12)*

*… it was quite easy if you look at the pamphlets and the sketches to see exactly what you had to do. (Ppt 15)*

#### Tailoring of the exercise programme

The provision of 1:1 support, and the flexibility for physiotherapists to personalise and recommend modifications to the exercise programme, including whether to use equipment like gym balls and TheraBands, the number of repetitions to execute, and omission of certain exercises, enabled the opportunity for the exercise programme to be tailored to a participant’s ability. This suggests that attempts were made to make the intervention fit with a person’s physical capability to reduce barriers to engaging in the activity.

…*we worked out what ones I could do and couldn’t do. […] it was basically looking in a booklet and saying*,* well [Ppt 17] can you do this and can you do that one and then she put crosses on the ones that obviously*,* she didn’t think I could possibly do*,* so. (Ppt* 17)

However, despite this tailoring, many participants said that they did not adhere to everything that they had been asked to do, with only a minority reporting that they had been able to perform all of the recommended exercises. Instead, they found various exercises in their programme to be very difficult to perform (e.g., coming up from sitting, bending down), beyond their physical capabilities, and they struggled to execute all of their exercises, or to reach the recommended number of repetitions. Consequently, several participants assumed a more tokenistic approach and reported that they did just ‘as much as they could’.

*I: And were you able to do as many repetitions as they recommended*,* or as often?*

*Ppt 14: Not quite*,* but I did what I could do and that was it.*

Participants reported a range of opinions regarding the acceptability of the taste, texture, and flavour of the protein supplements. For example, whilst the shakes were enjoyable for some, others described them as unpleasant and grainy, and one participant reported that they had made them feel nauseous. However, because participants had the option of two different types of protein supplements and a choice of flavour options for each, they were able to find the supplement that was most palatable to them, albeit sometimes after a process of trial and error.

*But I had the jellies. I had the orange and the red one*,* but I didn’t like the orange ones at all. They really went against me. But the red ones*,* I think it was fruit cocktail or something. I had that. It was quite nice. Well*,* not bad. But I didn’t like the other ones. (Ppt 14)*

Participants also tailored how they consumed the supplements to increase their enjoyment and/or acceptability. For example, eating the jelly with ice cream or straight from the fridge. This, together with the flexibility to request an alternative supplement if required due to pragmatic reasons (e.g. going on holiday), influenced participants perception and experiences of the intervention.

*… you didn’t have to put them in the fridge*,* but I found I used to put three or four in the fridge because I found they were more enjoyable to eat after being in the fridge than when it was not. (Ppt 12)*

### Theme 2: Internal factors

Several internal factors influenced participants willingness and ability to engage with the recommended exercises and/or consumption of the supplement.

#### Pain

Pain underpinned participants experiences and perception of the exercise intervention. Most lived with chronic background pain in the affected hip or knee joint, and some also suffered from pain elsewhere in their body. Several described how pre-existing pain had made it harder to attempt some of the exercises, but also how the exercises aggravated and intensified their pain.

*I struggle with most of them actually […] I have the pain in the one hip and in the knee of the other and my shoulders are not good. I’ve got tears in the tendons and everything. So you know*,* my arms are not good to use as well. (Ppt 14)*

*I: Did you notice any kind of benefits from doing [the exercises]?*

*Ppt15: No*,* not one bit. In fact*,* they made [the pain] worse […] They just get harder actually for me […] 20% I could do. The rest was just too painful mainly.*

They implemented strategies to help cope, such as reducing the number of repetitions carried out of each exercise and taking rest breaks in-between sets. However, despite this, the pain experience negatively impacted on the willingness and ability of some participants to adhere fully to the recommendations, and for a small number it meant they stopped altogether. Pain therefore limited participants capability to adhere to the recommendations.

#### General health and wellbeing

Minor illnesses, such as colds and sickness bugs, affected participants ability and willingness to engage with both the exercise programme and protein intervention, and meant that occasional days were missed, even by those who had adhered well to recommendations, when feeling well. More serious health issues entailing hospital stays, meant that one participant was unable to engage in the interventions for a lengthy period whilst recovering, and for another the interruption led to them choosing to disengage altogether from the interventions.

*… I had to go in and have this kidney stone removed and then I developed an infection […] when I came home I just had to get over everything that had gone on […] when I started to feel better*,* what I did find though because I’d had that gap and I was quite weak from everything that had gone on*,* I did have to build up again*,* I couldn’t go straight back into doing a full routine.(Ppt 13)*.

Due to co-morbidities, two participants reported long-standing issues with swallowing, which impacted on their experience of, and engagement with, the protein intervention. Although for one the struggle to swallow the protein supplements led to a decision to stop taking them before the end of the 12-weeks, for the other it only influenced her decision to choose the jelly over the shakes as they were seen as a more viable and acceptable option.

*I: Did you consider having the protein shakes?*

*Ppt 18: No because I’m not very good with swallowing*,* if it was something I didn’t like I wouldn’t have been able to take it. You know*,* I thought I’d be okay with the jellies […] All I can think of is when I had to have something*,* oh an X-ray at some point I had to take that barium*,* you know*,* that just goes through my mind when I*,* you know*,* when you think of you know drink.*

#### Personal motivations and perceived value

Internal drive to do the best for themselves and to engage in behaviours that could help to improve their situation, both during the pre- and postoperative period, motivated some participants to undertake the exercise recommendations, despite their pain experience and the challenges of doing so. Behaviour was driven by various beliefs, including the view that they would not be being asked to undertake the exercises if there was no value to them and the perception that they would be ‘cheating’ themselves if they did not engage properly.

*I thought well if it’s going to help*,* I’ll do anything […] I sort of persevered and got them done because I thought*,* well they wouldn’t tell me to do them if it isn’t going to help me. (Ppt 12)*

Some participants reported experiencing psychological (for example, feeling more energised) and/or physical benefits (for example, able to move freer and with more ease) during the intervention period, which they attributed to undertaking the exercises.

*I found it actually believe it or not helped my head clear […] I was able to move freer […] I found it easier getting up and down the stairs. Yes*,* moving around basically […] I’ve felt much better. (Ppt 7)*

Many participants considered that supplementing their diet with additional protein could have potential benefits to their health and wellbeing. However, when questioned, only one participant credited the changes they had observed (improved mood and feeling better) to taking the supplements.

*And I felt it was doing me good […] I felt better on the protein than when I don’t […] I felt life was a little bit happier*,* even though I was in pain. (Ppt 19)*

The belief that carrying out the recommended behaviours may offer benefits to themselves and/or observing benefits, could have influenced motivation to engage with the recommendations.

### Theme 3: Environmental/external influences

Factors external to participants also influenced their willingness and ability to enact the recommended behaviours.

#### Social influences

Although participant’s social support networks did not appear to be involved in, or influence, their experience with the protein intervention, some indicated that other people had positively affected their engagement with the exercise recommendations. Immediate family members were a source of emotional (e.g., offering encouragement) and practical (e.g., helping with the practicalities of certain exercises, or inflating the exercise ball) support.

*I: Did your family support you with doing the exercises at any time?*

*Ppt 14: Yeah*,* the one daughter. She would always say ‘do the exercises’. […] she just made sure that I was doing them [laughs]. I’ve got to do as I’m told. She’s the bossy one.*

#### Daily life: integration, interruption and changes to usual routine

The majority of participants said they had integrated their exercises into daily life, and rather than having a fixed time of day when they would undertake the regimen, they chose to make the routine fit in with, or around, their plans for the day. Some also spoke of splitting their routine into two to facilitate this.

*Well I tried to work it around whatever I was doing that day. I’d do say half the exercises in the morning and then later in the day I’d do the other half. (Ppt 7)*

Those participants who reported not adhering to the recommendation to take the protein supplement within 3-hours of undertaking the exercise programme, preferred to take it instead at a time of day that was more convenient to them, for example with their evening meal even if the exercises had been undertaken in the morning. Choosing to take a more flexible approach to both elements of the intervention, may have helped to increase acceptability and limit the burden of undertaking the requested behaviours, encouraging more sustained engagement.

However, other commitments and changes to normal routine, including pre-planned social activities and holidays could act as a barrier to undertaking the exercises and taking the protein supplement on the effected days. For some, this meant occasional days were missed as they didn’t have the time or simply ‘forgot’. For others, a break from usual daily life resulted in a lack of engagement for more prolonged periods.

*I: …so how long did you have a break from the protein for then?*

*Ppt 16: Two weeks*,* and then I had the jellies when I came back again.*

For one participant however, this interruption and break to the routine of their exercise regimen whilst on holiday, led to a shift in their attitude towards the intervention, and after returning home they struggled to re-engage, eventually choosing to stop altogether.

*I came back off holiday*,* it was getting back into it again I struggled with. And I did that for a few weeks*,* and then I think it wasn’t because what’s the point of this at all*,* but I just*,* oh I’ll do it tomorrow […] then I was doing it every other day or something and that’s really pointless. So*,* that’s when I came to a stop […] I hadn’t done it for a fortnight*,* so why have I got to rush back into doing it*,* that type of stupid attitude. (Ppt 17)*

## Discussion

This qualitative study generated important insights into the barriers and facilitators that played a role in influencing older frail patients’ willingness and ability to engage in a multimodal prehabilitation programme while waiting for THR or TKR. These factors were conceptualised as being specific to the design of the intervention, as internal to the participant (e.g. psychological factors such as motivation as well as physical factors including pain and illness) and as external factors/influences (such as life events and social support). Key facilitators to engagement included a 1:1 face-to-face physiotherapy appointment, regular follow-up telephone support, provision of clear written information, tailoring of the programme to individual capability, the perception that the programme would be beneficial, family support and integration into daily routine. Barriers were that the exercises were often too difficult to perform, joint pain, other illnesses and commitments, and a lack of motivation.

The need to incorporate the early identification of frailty and provision of tailored interventions into orthopaedic care is acknowledged, as frail patients often wait the longest for surgery and are most at risk of poor outcomes [29]. Despite this, a national survey of current NHS practice found a lack of tailored prehabilitation provision for frail patients [30]. Waiting lists for joint replacement are long and hospitals have different strategies to reduce waiting lists; independent sector treatment service providers or short stay pathways often discriminate against frail patients and exacerbate health inequalities, with healthier patients chosen for these pathways. Furthermore, deprivation, Asian ethnicity, older age, and female sex increase the risk of frailty, further increasing health inequalities [31].

Exercise adherence in older people is multifactorial, involving demographic, health-related, physical and psychological factors [23]. Our study identified barriers and facilitators that are applicable across different patient populations and clinical contexts. For example, the importance of psychological, social and environmental factors; physical health; motivation and perceived health benefit [32–35]. However, we identified important barriers and facilitators that are specific to older frail adults. A key theme that emerged from the current study that is specific to frail patients is the importance of tailoring exercise within prehabilitation programmes to the capability of older adults with frailty. This finding is consistent with a qualitative study exploring home-based exercise plus nutritional advice for older people with frailty preparing for cancer surgery [36]. If the exercises are perceived to be beyond their capabilities, then people are less likely to attempt them or maintain ongoing engagement. Standardised exercise programmes developed for the general population of patients undergoing joint replacement are unlikely to be suitable for all frail patients. Therefore, this needs to be considered in the design of prehabilitation programmes to mitigate against barriers to engagement in prehabilitation for frail patients. For example, through providing different levels of exercise and options for equipment-free exercises. Another key finding from this study and the study by Barnes et al. [36] was the importance of appropriate support and provision of a safe environment to practice the exercises and build confidence. This suggests that fully home-based programmes or digital interventions may impose barriers for this patient population. The implications of these findings for a future RCT are considered more fully in the article presenting the feasibility study findings [25].

To the best of our knowledge, this is the first study that has explored frail older adults’ experiences of engaging in an exercise and protein programme whilst waiting for THR or TKR. By using qualitative methods, we were able to gain valuable insight into the range of barriers and facilitators that influenced adherence to the intervention. These understandings enabled the identification of which elements of the intervention were helpful or unhelpful in facilitating behaviour change and identification of important recommendations to help shape future prehabilitation programmes within this population. In addition, the active involvement of a PPI group informed the topic guide and helped shape and validate the key themes identified within our work. However, limitations of this research need to be considered when interpreting the results. Firstly, as the recruitment target for the embedded qualitative study related to the larger group of randomised participants (i.e. those allocated to both the usual care and intervention groups), it is possible that we did not reach data saturation. Therefore, new themes relating to the experiences of undertaking the intervention may have emerged if we had been able to carry out additional interviews with this sub-group. It is a possibility that the experiences of the 10 participants in our study, all of whom were people willing to participate in a randomised feasibility study of prehabilitation, may not be reflective of the wider target patient population. In addition, although participants were recruited from three NHS hospitals in England and Wales to allow for geographic diversity, everyone who agreed to the interview in the intervention group identified as White British or Welsh. Future work needs to ensure the involvement of older frail patients from underserved ethnic minority communities. This will help us to understand if, and how, their experiences of prehabilitation differ to those involved in this study.

It is worth noting, that all but one of the intervention group participants requested for the interview to take place over the telephone. Although this mode of data collection offers some advantages over in-person interviews such as convenience, accessibility and affording a sense of anonymity which can encourage more openness, there are also limitations. These include challenges associated with the loss of nonverbal and visual cues and potential difficulties in establishing rapport [37]. The interviewer took steps to mitigate these limitations where possible. This included: engaging the participant in a more general chat before initiating the formal interview to build rapport, providing verbal feedback and reassurance, engaging in active listening and paying close attention to subtle changes in tone and pacing in the absence of nonverbal cues. Finally, viewing the results through a theoretical lens could have provided additional insights to further enhance understanding of engagement with the intervention. Future work in this area could draw on health psychology models and frameworks to facilitate this.

This study has generated important insights to inform the design of prehabilitation programmes for frail patients waiting for joint replacement. Through embedding qualitative research within a feasibility study, we generated insight into key barriers and facilitators to engagement in prehabilitation, some of which were unique to the older frail population, and identified important design considerations for prehabilitation programmes to improve accessibility and adherence, prior to a RCT. Providing tailored prehabilitation to frail patients undergoing THR/TKR is a novel approach to optimising outcomes for a group of patients often underserved in research and healthcare, and evaluation of the effectiveness of this approach is needed to optimise health, reduce surgical complications and address increasing healthcare costs.

## Conclusion

In conclusion, our study identified multifactorial barriers and facilitators, which relate to intervention design, internal factors and environmental/external influences, that effect frail older adults’ adherence to multimodal prehabilitation before joint replacement. While many barriers and facilitators experienced by frail patients are consistent with those experienced by non-frail patients, our study highlights the importance of tailored prehabilitation programmes which provide additional support and resources to optimise accessibility and adherence.

## Supplementary Information

Additional file 1. COREQ_Checklist.

Additional file 2. Interview Topic Guide.

## Data Availability

Data are available in a public, open access repository. The datasets generated from this study will be available in the University of Bristol Research Data Repository. Data will be available within 6 months following publication of the feasibility study findings. Access to the data will be restricted to ensure that data are only made available to bona fide researchers for ethically approved research projects, on the understanding that confidentiality will be maintained and after a data access agreement has been signed by an institutional signatory.

## Abbreviations

RCT: Randomised controlled trial
THR: Total hip replacement
TKR: Total knee replacement
NHS: National Health Service
PEP-R: Patient Experience Partnership in Research group
GFI: Groningen Frailty Indicator
