# Supplementary material for: Multimodal prehabilitation for frail older adults having hip or knee replacement: a qualitative exploratory study of barriers and facilitators
Source: BMC Musculoskelet Disord. 2026 Apr 20;27:472. doi: 10.1186/s12891-026-09803-z (PMC13227803; doi:10.1186/s12891-026-09803-z)
Supplement: Supplementary file 2 — Additional file 2. Interview Topic Guide. [file 12891_2026_9803_MOESM2_ESM.pdf]

Interview topic guide for Joint PREP randomised participants

Topic guide to be used flexibly. Interviewer will use probes and follow-up questions where appropriate.

1. **Background and introduction**

- To start off, please could I ask for some information from you? We ask these questions just to make sure that we are speaking to people with different characteristics.

    - ethnic group
    - educational level
    - employment status
    - co-morbidities
    - live alone/with others
    - Age

- Confirm that participant agreed to be randomised to the Joint PREP trial. Check treatment allocation and whether they have had surgery yet.

- If we could think now about your journey towards your hip or knee replacement surgery. Are you able to tell me a little bit about when your problems first began?

- How do other people support you?

- Do you have any experience of accessing physiotherapy services? If yes, explore experiences of physiotherapy and attitude towards.

- Have you been offered any equipment to help you around your home to help you manage better, e.g., shower chair, additional handrails?

- Have you taken part in any research studies before?

2. **Views on taking part in an RCT**

- I'd like to talk to you now about when you first heard about the Joint PREP trial. Can you tell me how and where you found out about it?

- What were your first thoughts about taking part?

- Can you tell me *why* you decided to take part?

- From the information that you received (i) what did you understand to be the purpose of the study, the reason it was being done? (ii) what was your understanding of what the study would involve for people who agreed to take part?

- Thinking both about the written information you were given about the trial and the conversations that you have had with staff members; (i) do you think you were given enough information about the different approaches to treatment as part of the trial? (ii) do you think you were given enough information or not about how the treatment was to be decided?

- Was there anything that you didn't understand about the study? Was anything not clear or confusing? Did you feel able to ask them questions?

- Overall, what do you think about the information that you have received about the Joint PREP trial?

- In addition to the information that you received about the study in the information booklet or through conversations with the doctors/nurses, have you sought out or been given any other information e.g., about exercises that may help you or on protein supplements? If yes, explore further.

- Have you spoken to others (experts/family/friends) or tried to find out any information on your problems with your hip/knee or what can help in other ways, for example, through your local library, media, internet etc?

- Once you agreed to take part in the study, when did you find out which study group you had been put into? (*Ascertain what the patient understands. If necessary, explain the study options and the random allocation).*

- Based on what you know about the study, what were your thoughts about being put into the study group you were (X) instead of the other (Y) group? What are your thoughts on the importance of patients in the treatment group compared to patients in the usual care group?

- Did you have any initial concerns about being involved in the trial?

- Do you still have any concerns now?

- Explore experience of questionnaire completion.

3. Experiences of the intervention (<u>for intervention group only</u>)

Note: when asking these questions, consider whether participant withdrew from the treatment early and shape accordingly

- Now I would like to talk to you a little bit about your experiences of the intervention. Ask participant to talk about their contact with the physiotherapist (i.e., in-person/virtual) and frequency of contact.

- Now can you describe how you have found doing the exercises that were given to you to do at home?

- Let's think now about your experience of the protein supplement. Confirm if took the jelly/drink. Can you describe how you have found eating the protein jelly/drinking the protein shakes with regard to the taste and flavour?

- Did you eat the protein jelly (or drink the protein shake) within 3 hours of exercising, or did it vary?

- What aspects of the Joint PREP intervention could be improved and how?

- <u>For participants who withdrew early from treatment</u>: What would have helped you to continue with the treatment for the full 12 weeks?

4. Closing (<u>all participants</u>)

- Is there anything else you would like to add about what we have talked about today?

- Thank participant for their time**.**
